# Patient Safety Education in German speaking countries: first successes and blind spots

**DOI:** 10.3205/zma001230

**Published:** 2019-03-15

**Authors:** Jan Kiesewetter, Sabine Drossard, Tanja Manser

**Affiliations:** 1 Klinikum der LMU München, Institut für Didaktik und Ausbildungsforschung in der Medizin, München, Germany; 2 Universitätsklinikum Augsburg, Klinik für Kinderchirurgie und Kinderurologie, Augsburg, Germany; 3 Fachhochschule Nordwestschweiz FHNW, Hochschule für Angewandte Psychologie, Otten, Switzerland

## Editorial

As the topic of patient safety has gained increasing scientific and public interest, the teaching of patient safety has gradually been integrated in medical education at many universities in recent years. The Committee for Patient Safety and Error Management of the German Association for Medical Education flanked this process three years ago with the Learning Objective Catalogue for Patient Safety in Undergraduate Medical Education [1]. 

Shortly afterwards, the idea arose to compile a special issue on patient safety education in German-speaking countries. In particular, the committee wanted to determine in which areas of patient safety education research, individual teaching projects or pilot projects exist. Naturally, with this special issue, it is not possible to present a complete survey of the German-speaking teaching scene on patient safety. Some of the works are included in regular editions of the Journal for Medical Education. Other manuscripts may be found in journals focusing specifically on educational issues in medical specialties. Nevertheless, it is interesting to note which goals and competencies found in the learning objective catalogue for patient safety in undergraduate medical education are addressed in the papers published here. Specifically, in which subject areas these are located, whether courses offered are elective or compulsory, how many students may benefit from the courses, whether they are taught mono or inter-professionally, and whether the courses are organized as separate events or affiliated with other subjects.

On the other hand, it is at least as important to note where there are blind spots, i.e. where learning objectives contained in the catalogue of learning objectives have not experienced (published) coverage in German-speaking countries. From this, it can be deduced what the reasons for this non-coverage might be and how this deficit could be counteracted locally and structurally as well as organizationally in higher education policy. 

In the articles published here, the authors cover subjects of patient safety that have been classified as essential in the learning objectives catalogue, such as Error, Blame, and Responsibility [2] as well as work on extent and epidemiology with the Hotspots Hygiene [3], [4], Drug Therapy Safety, and Polypharmacy [5]. In a comprehensive course at the University of Marburg, these patient safety topics are supplemented with an additional topic: sources of medical errors in diagnosis and indication status. Also, adverse events or critical incidents and related organizational factors, are embedded as learning objectives in two courses [2], [6]. 

In two survey studies on the subject of interfaces, desiderata are formulated to improve handovers [7] and to teach and test them increasingly in an interprofessional context [8]. Initial approaches to implementing the above within the scope of medical education can be found elsewhere [9]. In addition, small work packages, deliverable by students, so called "entrustable professional acitivities" for students in the practical year were examined [10].

Within the scope of teaching strategies for patient safety, documentation and existing standards may be dealt with in courses on quality management; no papers on this topic where submitted to the special issue. In two research projects, [6], [11] students were sensitized to the recognition of occurred harm within a treatment and trained in the identification of factors that increase the likelihood of errors. In addition, an innovative teaching approach to interprofessional nutrition management was published. This approach serves as a link between teaching and clinical care, achieving a wide range of teaching objectives [12]. 

Despite the very broad spectrum of works within the complex topic of patient safety, papers were not submitted in all of the categories listed in the learning objective catalogue. In particular, it is noticeable that there were no submissions on projects related to learning objectives for electronic and mobile devices and human-machine interaction. This is of particular concern in relation to the strongly promoted digitalization in the health care system. This could be due to the fact that the studies or teaching examples were published elsewhere, or teaching units might be so new that publishable data is still missing. It seems important that if medical students are not brought closer to deal with the possible sources of error in digital health care, new risk potentials are created. Conversely, this development offers the opportunity to develop teaching units that impart both knowledge on the subject of patient safety and professional content knowledge. 

The articles published here do not focus on learning goals that relate to the individual physician. However, the limits of human performance capacity, the assessment of one's own competence, and the disclosure of one's own mistakes in the sense of "open disclosure" represent great potential for teaching innovations. As part of the Master Plan Medical Studies 2020, there are already initial approaches to communicating errors in a targeted manner [13]. 

Educational modules that convey knowledge about one's own stress factors as well as how to deal with them and the associated emotions could usefully supplement these teaching units. To teach medical students how to deal with stressful situations, and to point out possible support, is also urgently required given that 59% of all physicians feel psychologically burdened by their work and 2-3% are to be classified as clinically depressed [14], [15]. Training concepts on this topic have recently been developed, but have not yet been researched regarding their effectiveness [16]. 

It is understandable that patient safety is covered incompletely in this special issue, as it is a relatively new teaching discipline not yet included in the curriculum as a mandatory subject. On the other hand, however, it is encouraging to see how many teaching projects have emerged in recent years in many different locations. We hope that the articles published here will inspire other scientists and curriculum developers to integrate patient safety into their teaching.

## Competing interests

The authors declare that they have no competing interests. 
